# Identification of a New B-Cell Epitope on the Capsid Protein of Avian Leukosis Virus and Its Application

**DOI:** 10.3390/cimb46060350

**Published:** 2024-06-12

**Authors:** Zui Wang, Lina Liu, Junfeng Dou, Li Li, Qin Lu, Xinxin Jin, Huabin Shao, Zhengyu Cheng, Tengfei Zhang, Qingping Luo, Weicheng Bei

**Affiliations:** 1 State Key Laboratory of Agricultural Microbiology, College of Veterinary Medicine, Huazhong Agricultural University, Wuhan 430070, China; wangzui@webmail.hzau.edu.cn (Z.W.); lili_0215@126.com (L.L.); luqin198909@126.com (Q.L.); jinxinxin@webmail.hzau.edu.cn (X.J.); 2 Key Laboratory of Prevention and Control Agents for Animal Bacteriosis, Ministry of Agriculture and Rural Affairs, Institute of Animal Husbandry and Veterinary, Hubei Academy of Agricultural Sciences, Wuhan 430064, China; luohuawushengna@163.com (L.L.); djf0825@163.com (J.D.); shhb1961@163.com (H.S.); zhenyu.cheng@dal.ca (Z.C.); tfzhang23@163.com (T.Z.); 3 Hubei Hongshan Laboratory, Wuhan 430064, China; 4 Department of Microbiology and Immunology, Dalhousie University, Halifax, NS B3H 4R2, Canada

**Keywords:** monoclonal antibody, avian leukosis virus, B-cell epitope

## Abstract

Avian leukosis virus (ALV) is an avian oncogenic retrovirus that can impair immunological function, stunt growth and decrease egg production in avian flocks. The capsid protein (P27) is an attractive candidate for ALV diagnostics. In the present study, a new hybridoma cell (1F8) stably secreting an anti-P27 monoclonal antibody (mAb) was developed. The mAb exhibited a high affinity constant (Ka) of 8.65 × 10^6.0^ L/mol, and it could be used for the detection of ALV-A/B/J/K strains. Moreover, a total of eight truncated recombinant proteins and five synthetic polypeptides were utilized for the identification of the B-cell epitopes present on P27. The results revealed that ^218^IIKYVLDRQK^227^ was the minimal epitope recognized by 1F8, which had never been reported before. Additionally, the epitopes could strongly react with different ALV subgroup’s specific positive serum and had a complete homology among all the ALV subgroups strains. Finally, a new sandwich ELISA method was created for the detection of ALV antigens, demonstrating increased sensitivity compared to a commercially available ELISA kit. These results offer essential knowledge for further characterizing the antigenic composition of ALV P27 and will facilitate the development of diagnostic reagents for ALV.

## 1. Introduction

Avian leukosis (AL) is an infectious neoplastic disease caused by avian leukosis virus (ALV), which leads to stunted growth, reduced egg production and immunosuppression in avian flocks [1,2]. ALV belongs to the *Alpharetrovirus* genus within the Retroviridae family and exhibits a C-type morphology [2]. According to interference of the viral envelope, host range and cross-neutralization patterns, ALVs are typically categorized into seven subgroups (A, B, C, D, E, J and K) within chicken populations [2]. The exogenous viruses, namely subgroups A, B, C, D, J and K, have the potential to induce various types of neoplastic diseases in chickens [2]. Although ALV-E is reported to be a low- or non-pathogenic endogenous virus, it can interfere with antigen or nucleic acid detection of the exogenous virus [3,4]. The most prevalent ALVs in commercial poultry currently include subgroups A, B, J and K. Notably, subgroup K is newly identified in local domestic chicken breeds in China [5]. The highly efficient vertical and horizontal transmission routes, coupled with the absence of effective vaccines and therapeutic interventions, have contributed to the global prevalence of ALV, resulting in substantial economic losses within the poultry industry [6]. Until now, the most effective way to prevent and control ALV infection was population eradication. Therefore, it is crucial to develop an effective method for the accurate detection of ALV [7].

The genome of ALV primarily consists of gag (which encodes the internal structural proteins of the virion), pol (which encodes RNA-dependent DNA polymerase) and env (which encodes the envelope glycoprotein). Capsid protein P27 (encoded by gag) and envelope protein Gp85 (encoded by env) are the major candidate antigens for ALV detection [8]. Gp85 has subgroup specificity, which is mainly due to its hypervariable regions (hr1 and hr2) and variable regions (vr2 and vr3) [8]. Therefore, it is mostly used for subgroup specific antibody detection. On the contrary, the conservation of P27 is remarkably high across all subgroups, with more than 96% sequence identity [9]. In addition, P27 accounts for over 30% of the entire viral protein, which indicates that P27 can be detected easier than other antigens [10]. Thus, P27 protein stands as the foremost preference in the preparation monoclonal antibodies (mAbs) for ALV antigen detection. MAbs against P27 have been generated in other studies [11,12,13,14,15], and these antibodies were used to identify two different B-cell epitopes (^181^PPSAR^185^ and ^193^CFRQKSQPDI^202^) [12,13]. The identified epitopes may be crucial for both differential diagnoses and vaccine development. For example, a recently developed vaccine consisting of multiple epitopes has demonstrated robust efficacy against ALV-J infection [16], and the utilization of new B-cell epitopes in peptide-ELISA exhibits enhanced sensitivity for detecting ALV-J antibodies compared to conventional serological techniques [17]. Moreover, the localization of viral protein epitopes holds immense significance in unraveling the antigenic structure and molecular intricacies of virus–antibody interactions.

In this study, a specific mAb targeting ALV P27 was obtained. The mAb was subsequently employed for the screening of B-cell epitopes on P27 using Western blotting and peptide-ELISA. In addition, it was used to develop a sandwich ELISA for ALV antigen detection. These findings will greatly facilitate the development of a diagnostic kit for ALV infections and may provide valuable insights into unraveling the antigenic structure of the P27.

## 2. Materials and Methods

### 2.1. Cell Lines, Viruses and Reagents

The cell lines DF1 and SP2/0 were preserved by our laboratory and were maintained in DMEM (Gibco, Waltham, MA, USA) supplemented with 10% FBS (Gibco, USA). ALV-J (SX-18-01), ALV-A (XTM-21-01), ALV-B (SZ-19-01) and ALV-K (SH-20-01) strains, obtained from neoplastic chicken tissue samples, were cultivated on DF-1 cell monolayers. Newcastle disease virus (NDV, TS09-C), Avian influenza virus (H9N2, DY0602), Fowl adenovirus serotype 4 (FadV4, HB1510), Infectious bronchitis virus (IBV, HB120), Infectious laryngotracheitis virus (ILTV, WG strain), Infectious bursal disease virus (IBDV, HB0719), *Salmonella* gallinarum (C79-3), *Escherichia coli* (ATCC 25922), *Pasteurella multocida* (C48-1), *Mycoplasma synoviae* (XY-1) and *Mycoplasma gallisepticum* (DY-1) were stored in our laboratory. NDV, AIV, FadV4, IBV, ILTV and IBDV were cultured in SPF chicken embryos. *M. synoviae* and *M. gallisepticum* were cultured in modified Frey’s medium at 37 °C with 5% CO_2_. *Escherichia coli*, *Salmonella* and *Pasteurella* were cultured in TSB at 37 °C. The BALB/c mice were acquired from the Hubei Animal Disease Control Center, Wuhan, China.

### 2.2. Expression and Purification of P27 Proteins

According to the sequence of p27, the specific primers (p27-F/R) were designed. The amplicon of P27 was inserted into pET-28a vectors to obtain pET28a-p27. Subsequently, the plasmid pET28a-p27 was transformed into Rosetta (DE3) (Huayueyang, Beijing, China), and expression was induced by 0.5 mM IPTG (Thermo, Waltham, MA, USA) at 16 °C for 24 h. Next, the cells were harvested and subjected to sonication on ice, followed by purification of the soluble products using Ni-NTA affinity chromatography columns (Thermo, USA). Finally, the purified P27 was analyzed using SDS-PAGE and Western blotting mediated by an Anti-6X His tag^®^ (ABclonal, Wuhan, China).

### 2.3. Characterization and Identification of Monoclonal Antibodies

The complete/incomplete Freund’s adjuvant (Sigma, Ronkonkoma, NY, USA) was fully mixed with P27. Then, the antigen was administered to 6-week-old female BALB/c mice at a dose of 50 μg per mouse through multiple subcutaneous injections administered on the neck and back every two weeks. A total of three immunizations were performed. Two weeks after the last booster, serum was collected to detect the antibody titers. The mice exhibiting higher levels of antibodies were chosen for shock immunization. After three days, the mice were humanely euthanized to harvest spleen cells, which were subsequently fused with SP2/0 cells. The fused cells were maintained in a suitable dose of hypoxanthine-aminoperin-thymidin (HAT) or hypoxanthine-thymidine (HT) (Sigma, USA) medium as required. When the cell colonies reached 1/3 of the ocular microscope field, the hybridoma supernatants were absorbed for antibody detection via indirect ELISA. The anti-P27 positive hybridomas were subcloned via limiting dilution for at least three rounds and subsequently expanded to enable cryopreservation.

The expanded hybridomae were inoculated into female 8-week-old BALB/c mice to prepare ascites fluid. MAbs were obtained after ascites fluid was purified using protein G Agarose (Beyotime, Shanghai, China). The mAb was identified using purified P27 protein and ALV-A, B, J, K virus through Western blotting and IFA, respectively. Specific P27 antibody levels were determined with the indirect ELISA. In addition, the subclasses of the mAb were identified using a Mouse mAb Isotyping Strip (Roche, Indianapolis, IN, USA) according to the manufacturer’s instructions.

### 2.4. Western Blotting

The protein and virus samples were subjected to a 10 min boiling step, followed by separation using SDS-PAGE, and then transferred to a Nitrocellulose (NC) membrane (Biosharp, Hefei, China). The membranes were blocked with 5% skim milk (BD, Franklin Lakes, NJ, USA) at 37 °C for 1 h and then incubated overnight at 4 °C with the mAb (1:500 dilution in TBST). Subsequently, HRP-conjugated goat anti-mouse IgG was used to incubate the cell membranes (ABclonal, China) for 1 h at room temperature. Lastly, after washing five times, the bound proteins were identified using DAB (Beyotime, China).

### 2.5. Indirect Immunofluorescence

DF-1 cells were placed in a six-well plate and exposed to different ALV strains, respectively. After 7 days post-inoculation, the cells were fixed with 4% paraformaldehyde for 30 min at room temperature. After permeabilizing with 0.25% Triton-X 100 for 30 min, the cells were blocked with 5% skim milk in PBS for 2 h at room temperature. Then, the cells were incubated with mAb at 4 °C overnight. Then, the washed cells were incubated with diluted FITC-conjugated goat anti-mouse IgG (1:200 dilution in PBST) (Thermo, USA) for 1 h at room temperature. Lastly, the labeled cells were visualized through an inverted fluorescence microscope.

### 2.6. Indirect Enzyme-Linked Immunosorbent Assay (ELISA)

ELISA plates (NEST, Beijing, China) were plated with 100 μL per well of purified P27 protein (0.5 μg/mL) or synthesized peptides (1 µg/mL) at 37 °C for 2 h. After washing, the plates were blocked with 5% skim milk in PBST for 2 h at 37 °C. Then, the plates were incubated using various dilutions of the mAb at 37 °C for 1 h. Next, the washed plates were incubated with HRP-conjugated goat anti-mouse IgG (1:4000 dilution in PBST) at 37 °C for 1 h. Lastly, a TMB solution was added for 15 min and the absorbance at 630 nm (450 nm) was quantified. The binding affinity of the mAb was assessed by calculating the association constant (Ka) using a previously established method described in 1987 [18].

### 2.7. Identification of the Antigenic Epitope Recognized by the mAb

To map the epitope recognized by the mAb, 8 over-lapping fragments were obtained via PCR using the primers listed in Table 1. The amplicons were cloned into pGEX-6p-1 vectors. Subsequently, the recombinant proteins were expressed in Rosetta (DE3) competent cells and identified via a Western blotting method mediated by the mAb and anti-GST-Tag mAb (ABclonal, China), respectively.

The accuracy of the epitope was further assessed through the design and synthesis of a series of peptides by the Shanghai Jietai Biotech Company (Shanghai, China). These peptides were then analyzed using peptide-ELISA, and the absorbance at 450 nm was compared statistically. In addition, the validation of the interaction between ALV-positive serum (ALV-A/B and ALV-J) and linear B-cell epitopes was conducted using peptide-ELISA.

### 2.8. Biological Information Analysis

Conservation analysis of the identified B cell epitopes in the different ALV sub-group strains was measured though multiple sequence alignments using DNASTAR MegAlign (DNASTAR, Madison, WI, USA). The secondary structure, antigenic index and surface probability of the identified epitope were analyzed using Protean software (version 7.1) (DNASTAR, USA). The 3D model of P27 was constructed utilizing the SWISS MODEL online server with the capsid protein structure (SMTL ID: 1d1d.1) serving as the template. The location of the linear B-cell epitopes was visualized using PyMOL software (Version 3.0.3) (Schrödinger, New York, NY, USA).

### 2.9. mAb-Based Sandwich ELISA

The purified 1F8 mAb (6.7 μg/mL) diluted in 0.1 M carbonate buffer was coated into a 96-well ELISA plate (NEST) at 4 °C overnight. After washing, the plates were blocked with 5% skim milk for 2 h at 37 °C. Subsequently, the plates were incubated using various viruses (NDV, AIV, FadV4, IBV, ILTV, IBDV, *M. synoviae*, *M. gallisepticum*, *Escherichia coli*, *Salmonella* and *Pasteurella*) or samples at room temperature for 50 min. Next, the washed plates were incubated with an HRP-conjugated rabbit anti-p27 polyclonal antibody (1:1000) at room temperature for 50 min. Lastly, a TMB solution was added for 15 min, and the absorbance at 630 nm was quantified.

## 3. Results

### 3.1. Expression and Purification of P27 Proteins

**Figure 1:**
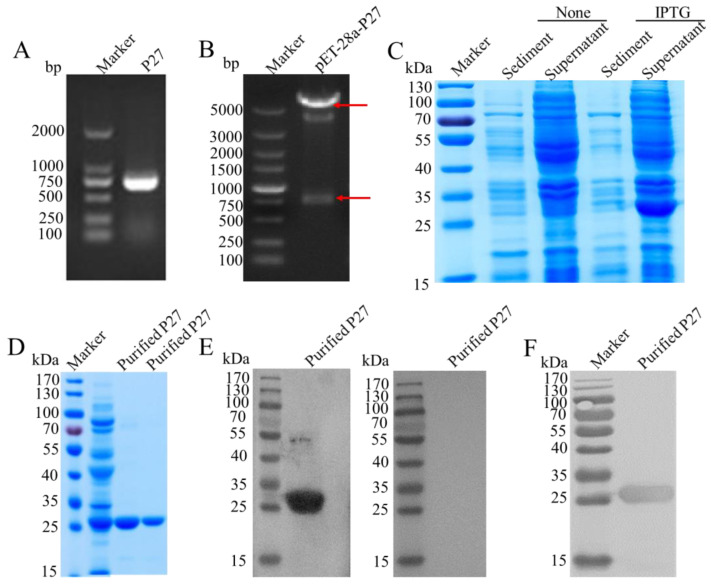
Preparation and identification of the ALV capsid P27 protein. (**A**) Identification of P27 amplicon. (**B**) Identification of the recombinant plasmids pET-28a-p27 via RE digestion (BamH I and Hind III). Identification on the expressed (**C**) and purified products (**D**) of P27 via SDS-PAGE. Western blot analysis of the purified P27 protein using (**E**) positive serum, negative serum and (**F**) an anti-His antibody. The red arrow indicates the presence of two bands after RE digestion.

The whole ALV p27 gene (720 bp, Figure 1A) was amplified from the proviral DNA extracted from the ALV-J (SX-18-01) strain and cloned into pET-28a to obtain pET28a-p27. pET28a-p27 was identified via sequencing and RE digestion (Figure 1B). Subsequently, it was transformed into Rosetta (DE3) and expressed successfully. The expressed products were found to have an expected size of approximately 26 KDa and were predominantly in a soluble form (Figure 1C). They were subsequently purified using Ni-NTA affinity chromatography columns and identified through SDS-PAGE (Figure 1D) and Western blotting using a His-Tag monoclonal antibody (Figure 1F) and positive chicken serum (Figure 1E).

### 3.2. Characterization and Identification of Monoclonal Antibodies

**Figure 2:**
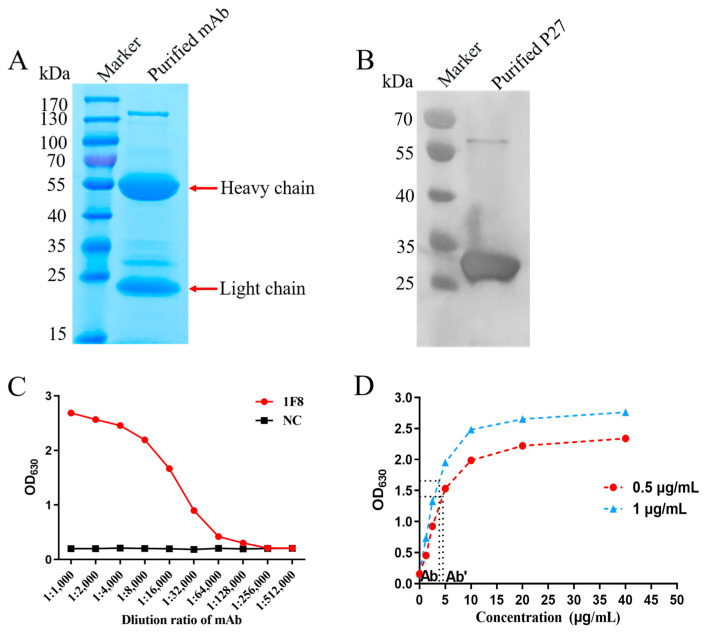
Preparation and characterization of the mAb. (**A**) Identification of purified 1F8 via SDS-PAGE. (**B**) The reactivity of 1F8 against P27 via Western blotting. (**C**) Indirect ELISA detected the titer of the mAb. (**D**) Binding affinity curve of the mAb.

To generate hybridoma cells that secrete specific monoclonal antibodies against P27, BALB/c mice were immunized with the purified P27 protein. One monoclonal antibody, called 1F8, exhibited the consistent production and secretion of antibodies targeting P27. For better follow-up experiments, the collected ascites samples were purified using the Caprylic acid-Ammonium sulfate method and identified. The result showed two distinct bands corresponding to the heavy and light chains at 55 kDa and 25 kDa (Figure 2A), respectively, indicating that highly pure monoclonal antibodies were successfully obtained. Western blotting analysis showed that the mAb can bind to the P27 protein under denaturing conditions, indicating its potential to recognize linear epitopes on the ALV-J P27 protein (Figure 2B). Moreover, the finding of subtype identification showed that mAb 1F8 was identified as subclass IgG1 with κ-type light chains, and its titer was detected as 1:1.28 × 10^5^ (Figure 2C). In order to assess the binding capability of the mAbs in conjunction with P27, the affinity constant determination curves of the mAb were generated (Figure 2D). Based on the formula from the previous study [18], the affinity constant (Ka) of mAb 1F8 was 8.65 × 10^6.0^ L/mol.

### 3.3. Specificity of mAbs

**Figure 3:**
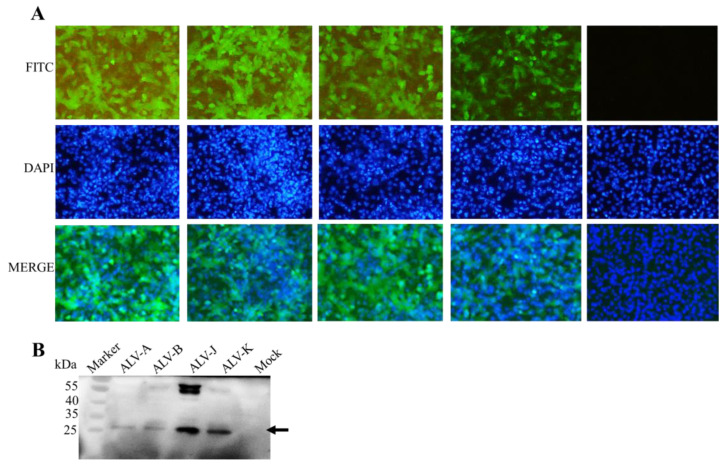
The specificity of the 1F8 mAb. The reactivity of 1F8 against different ALV subgroups strains using (**A**) IFA (100× magnification) and (**B**) Western blotting. IFA (FITC = green); DAPI (nuclear stain = blue). The black arrow points to the specificity reaction band.

To analyze the specificity of the mAb 1F8, Western blotting and IFA were employed to confirm the reaction between the mAb and four different subgroup strains (Figure 3A). The results of Western blotting showed that mAb 1F8 could react with the P27 of ALV-A, B, J and K strains. Results from IFA confirmed that the DF1 cells infected with ALV-A, B, J and K strains exhibited the manifestation of green fluorescence (Figure 3B). These findings indicated that the prepared monoclonal antibody had the ability to identify the four predominant ALV subgroup strains found in China’s commercial poultry industry.

### 3.4. Identification of the Antigenic Epitope Recognized by the mAb

**Figure 4:**
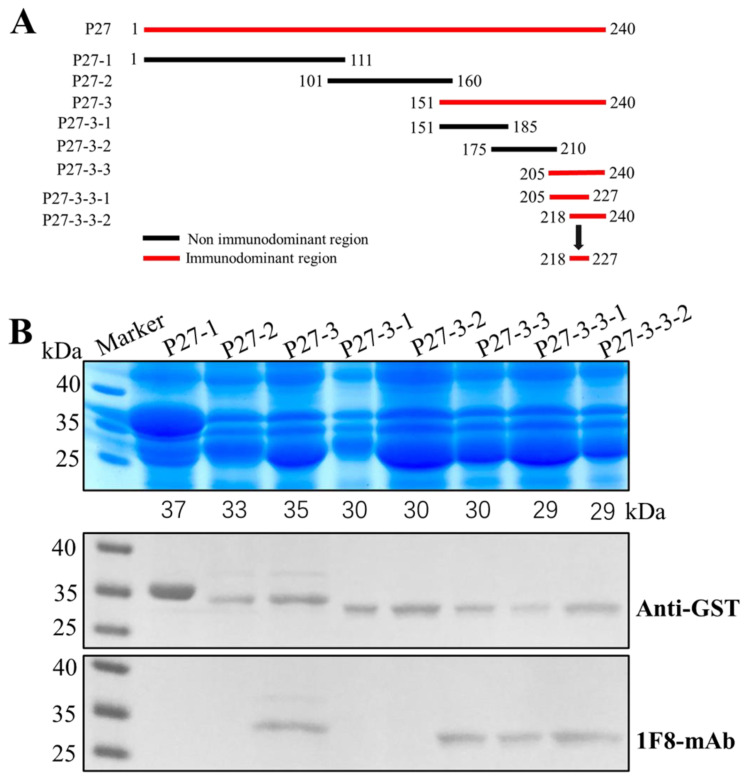
Detection of the B-cell epitopes of ALV P27 by mAb 1F8. (**A**) Schematic diagram for ex-pressing different ALV P27 truncations. (**B**) Detection results of eight truncated segments via SDS-PAGE and Western blotting mediated by the anti-GST and 1F8 mAb.

In order to map the linear B-cell epitopes on that ALV-J P27 protein that were recognized by mAb 1F8, three truncated and overlapping GST-fused P27 fragments, named P27-1 (1–111 aa), P27-2 (101–160 aa) and P27-3 (151–240 aa), were successfully expressed in *E. coli*. As illustrated in Figure 4A,B, among the three truncated proteins, only P27-3 could react with 1F8 mAb. This suggested that the epitope recognized by 1F8 was situated within 151 and 240 aa of the ALV-J P27 protein. Subsequently, the overlapping region of P27-3 was truncated into three segments and expressed with a GST-tag, including P27-3-1 (151–185 aa), P27-3-2 (175–210 aa) and P27-3-3 (205–240 aa). Among the three truncated proteins, only P27-3-3 could react with the 1F8 mAb, which indicated that the epitope recognized by 1F8 was positioned within 205 to 240 aa of the ALV-J P27 protein. Furthermore, the overlapping region of P27-3-3 was truncated into two segments and expressed with a GST-tag (P27-3-3-1, P27-3-3-2). The results of Western blotting showed both P27-3-3-1 and P27-3-3-2 exhibited reactivity with mAb 1F8, implying the presence of an antigenic epitope from position 218 to 227 aa.

**Figure 5:**
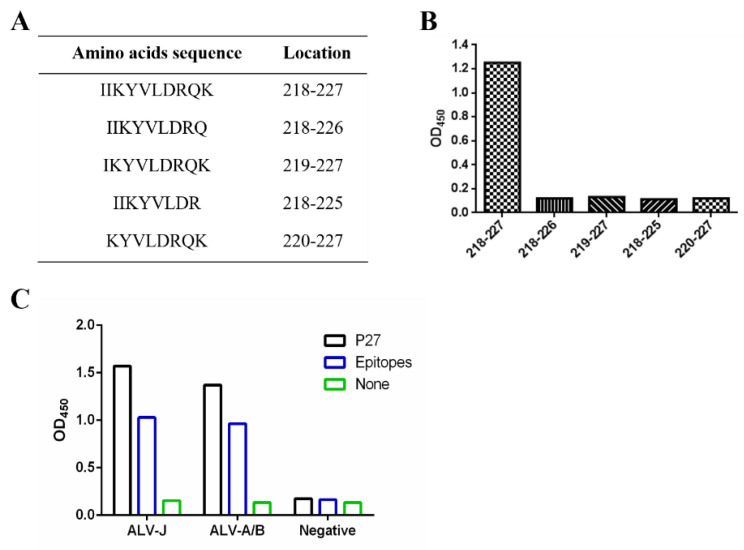
Identification of the minimal B-cell epitope using peptide-ELISA. (**A**) Peptides synthesized for epitope validation. (**B**) Reaction of 1F8 with the truncated peptides. (**C**) Reactivity of the identified epitopes ^218^IIKYVLDRQK^227^ with ALV-positive serum (ALV-A/B and ALV-J) and negative serum.

To further determine the minimal residues of the epitopes, the peptides ^218^IIKYVLDRQK^227^ were artificially synthesized and truncated from their N- and C-termini (Figure 5A), and indirect ELISA was used to screen these truncated peptides. As shown in Figure 5B, the optical densities under 630 nm (OD630) of the peptide containing the complete ^218^IIKYVLDRQK^227^ were much higher than those of truncated peptides. All data obtained above indicated that ^218^IIKYVLDRQK^227^ was an optimal liner B-cell epitope on the ALV-J P27 protein. Furthermore, the interaction between the liner B-cell epitope and ALV-positive serum was performed via peptide-ELISA. As illustrated in Figure 5C, the epitopes ^218^IIKYVLDRQK^227^ could be strongly recognized by ALV-A/B and ALV-J subgroup-specific positive serum, which suggested that the epitopes could possess the ability to stimulate a humoral immune response and might be used for the antibody detection of ALV.

### 3.5. Spatial Structures and Biological Characteristics of the B-Cell Epitopes

**Figure 6:**
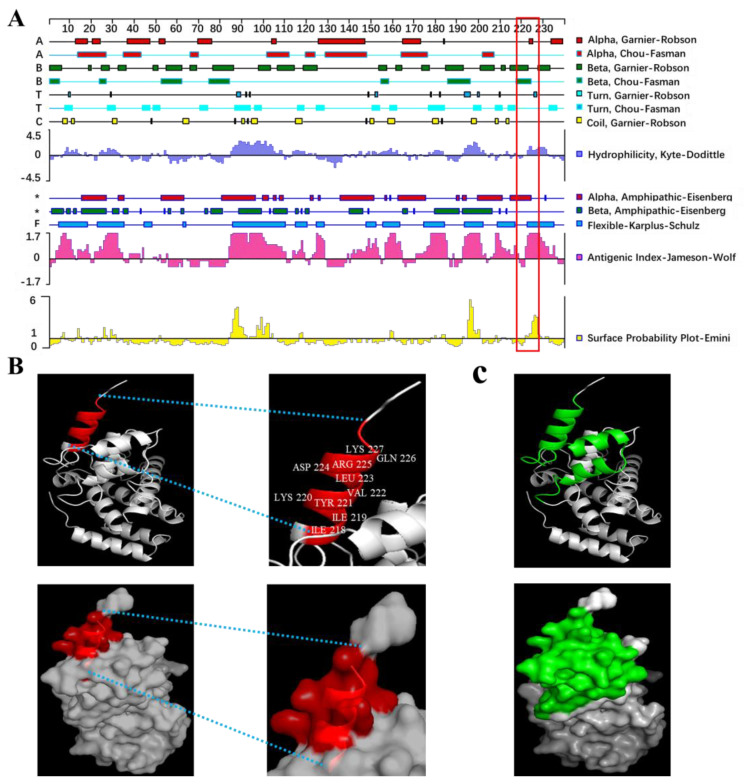
Prediction based on antigenic characteristics (**A**) and the spatial structures (**B**) of the mAb’s antigenic epitope. The epitopes ^218^IIKYVLDRQK^227^ are highlighted in red or red box. (**C**) Spatial structures of a prominent B-cell epitope domain (181–227 aa). The domain is highlighted in green.

In order to enhance the understanding of the spatial configurations associated with the antigenic epitope recognized by 1F8 mAb, DNASTAR Lasergene (version 17.3) was used to analyze the secondary structure, antigenic index, surface probability and 3D structure of the epitopes. As shown in Figure 6A, the epitope contained one α-helix based on Garnier–Robson prediction and one β-sheet based on Chou–Fasman and Garnier–Robson prediction and had a turn and a flexible region. In addition, the epitope was found to be situated on the outer surface of P27, exhibiting a significant antigenic index and pronounced hydrophilicity. Therefore, the results indicated that this region had a high possibility of epitope formation and was easier to be chimeric with antibodies because of its flexible and easier twisting and folding. Furthermore, PyMOL software was used to analyze the 3D model of the P27 protein (Figure 6B). Cartoon diagrams of the P27 protein view showed that the epitopes were exposed on the surface, and most of them were located in an α-helix, except Lys 227. In total, the epitope ^218^IIKYVLDRQK^227^ on the P27 protein of ALV-J was suggested to be an important B-cell epitope.

### 3.6. Conservation Analysis of the Epitope P27 in Different Strains

**Figure 7:**
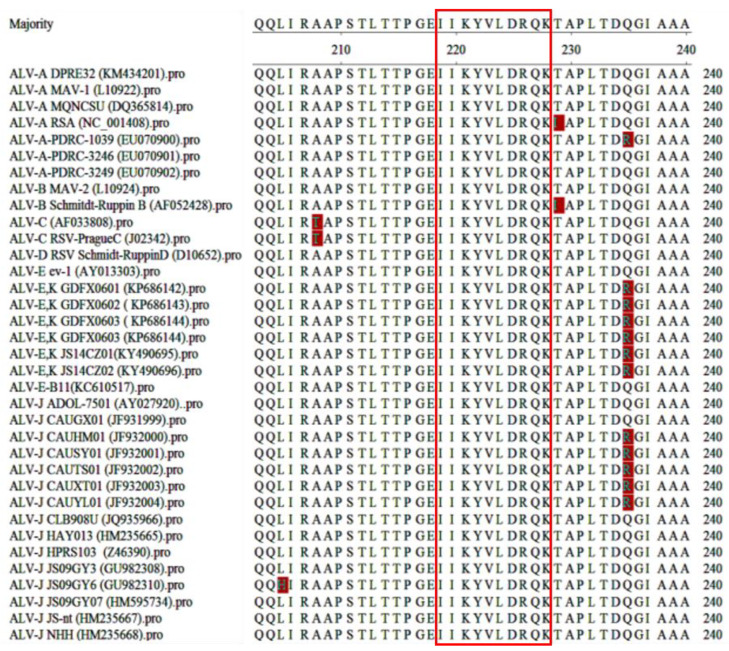
Conservation analysis of the B-cell epitope in different ALV subgroup strains. The epitopes ^218^IIKYVLDRQK^227^ are highlighted in a red box.

To assess the conservation of the identified epitope across various ALV strains, we selected many P27 protein sequences from different subgroups available in GenBank. These sequences were subjected to phylogenetic analysis and epitope comparison using MegAlign software (version 8.1.4). As depicted in Figure 7, the epitope ^218^IIKYVLDRQK^227^ exhibited a high degree of conservation among diverse ALV strains with no amino acid site differences.

### 3.7. Primary Application of 1F8 mAb in Serological Approaches for ALV Detection

**Figure 8:**
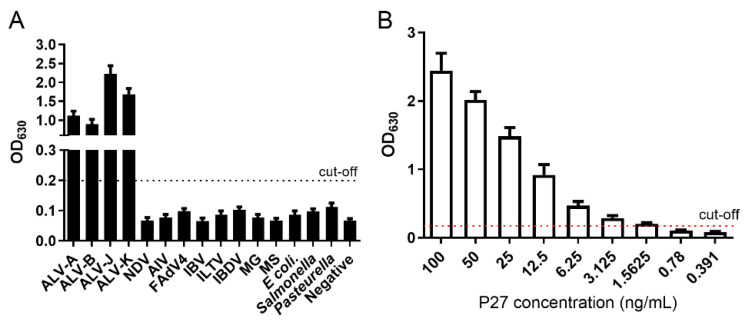
Establishment of a sandwich ELISA using 1F8 mAb. (**A**) The specificity of the sandwich ELISA was analyzed using different avian viruses and bacteria, including NDV (10^6.0^ EID_50_/100 μL), AIV (10^5.0^ EID_50_/100 μL), FadV4 (10^5.0^ EID_50_/100 μL), IBV (10^4.0^ EID_50_/100 μL), ILTV (10^4.0^ EID_50_/100 μL), IBDV (10^3.0^ EID_50_/100 μL), *M. synoviae* (10^8.0^ CCU/100 μL), *M. gallisepticum* (10^8.0^ CCU/100 μL), *Escherichia coli* (10^8.0^ CFU/100 μL), *Salmonella* (10^8.0^ CFU/100 μL) and *Pasteurella* (10^8.0^ CFU/100 μL). (**B**) Sensitivity of the sandwich ELISA was analyzed using the diluted P27 (100, 50, 25, 12.5, 6.25, 3.125, 1.5625, 0.78 and 0.391 ng/mL). The cut-off value was 0.2.

To determine the potential use of mAb 1F8 in developing a serological approach for ALV detection, a sandwich ELISA was established using the mAb 1F8 and HRP-conjugated rabbit anti-p27 polyclonal antibody. The cut-off value was set at 0.2, defined as the mean value plus a threefold standard deviation of negative chicken serum determined via IDEXX ELISA. The sandwich ELISA specifically reacted with ALV-A/B/J/K subgroup strains, but not with other common avian pathogens (Figure 8A). Sensitivity analysis demonstrated that the sandwich ELISA had a limit of detection (LOD) of 1.56 ng/mL P27 (Figure 8B).

To assess the performance of the sandwich ELISA, various clinically positive samples (albumen, plasma, semen and cloacal swabs) were detected using both the sandwich ELISA and IDEXX ELISA methods. The LOD for ALV detection in plasma, semen and cloacal swabs using the sandwich ELISA was found to be 2–4 times higher compared to that of the commercial kit (Table 2). In addition, a total of 460 albumen and 184 cloacal swabs were detected by the two ELISAs. As shown in Table 3, the sandwich ELISA exhibited excellent agreement with the IDEXX ELISA for detecting albumen samples (452/460 = 98.26%), while the coincidence rate was 90.76% (167/184) for detecting cloacal swabs. In order to further verify the specificity of 17 cloacal swabs with differences detected using the two methods, the samples were retested via qPCR. The results revealed that 15 cloacal swabs tested positive in the qPCR assay, whereas 14 were positive in the sandwich ELISA, and only 3 showed positivity in the commercial ELISA. The sandwich ELISA has a higher positive detection rate of ALV in cloacal swabs.

## 4. Discussion

Several prior epidemiological findings have revealed the global occurrence of ALV infection [19,20,21]. As a retrovirus, ALV possesses the capacity to generate genetic diversity and undergo rapid evolution due to its error-prone RNA polymerase and frequent recombination events [22,23,24], which makes the prevention and control of the virus much more challenging. The most effective method currently to manage ALV infection lies in the implementation of population eradication. Given the pivotal role of capsid protein P27 as a primary candidate antigen for ALV detection, it is essential to gain a comprehensive understanding of the structural and antigenic properties of the P27 protein to facilitate an accurate differential diagnosis and pave the way for effective disease eradication in the foreseeable future. The present study focused on the generation and characterization of mAbs specifically targeting ALV P27, thereby providing new insights into epitope mapping of the ALV P27 protein.

In the present study, the P27 protein was expressed in a highly soluble form using the prokaryotic expression plasmid pET28a-P27 (His-tag). The purified P27 was used to immunize mice and generate splenocytes that produce antibodies. Subsequently, the obtained splenocytes were employed to derive hybridoma cells capable of secreting antibodies according to a previous study [25]. Then, a newly developed monoclonal antibody, named 1F8, was successfully generated to target ALV P27, and the titer of the mAb was 1:1.28 × 10^5^. The ALV-A/B/J/K strain could be detected using Western blotting with the prepared mAb. In addition, IFA results revealed that the mAb could specifically recognize ALV-A/B/J/K particles in infected DF-1 cells. Unfortunately, we were unable to confirm the specificity of mAb for ALV-C/D/E strains in this study because the strains had not been isolated in our lab to date. In addition, given the ongoing emergence of new ALV subgroups or mutant strains, there are many experiments about the specificity of the new mAb awaiting future study.

The epitopes, as pivotal antigenic components of viral structural proteins, possess the remarkable ability to elicit humoral immune responses against viruses [26]. Identifying B-cell epitopes in viral proteins holds significant meaning for vaccine development, clinical diagnosis and antibody production. [27,28,29]. Clearly defining epitopes targeted by monoclonal antibodies will greatly contribute to understanding antigen structure and advancing antiviral therapies [30,31]. Until now, various approaches have been devised to detect linear B-cell epitopes, such as utilizing phage-ELISA and software-based prediction techniques [32,33,34]. Due to the limitations of technology in our lab, the newly prepared mAb 1F8 was used for identifying antigenic epitopes of P27 proteins of ALV via Western blotting and peptide-ELISA in the present study. The findings revealed that the antigenic epitopes in the region of the P27 protein from amino acid ^218^IIKYVLDRQK^227^ consisted of a minimum of ten amino acids. Epitopes could be classified into two distinct categories: linear epitopes, also known as continuous epitopes, and conformational epitopes, which are alternatively referred to as discontinuous epitopes [35]. In the present study, mAb 1F8 was capable of reacting with the denatured P27-3-3-1 and P27-3-3-2, suggesting that the epitopes ^218^IIKYVLDRQK^227^ were linear. Moreover, the epitopes also exhibited strong reactivity with different ALV subgroup-specific positive sera, suggesting that chickens affected by ALV display an immune response specifically targeting this linear epitope.

Early reports had revealed two B-cell epitopes on the capsid protein of ALV, ^181^PPSAR^185^ [12] and ^193^CFRQKSQPDI^202^ [13], respectively, while the epitope recognized by 1F8 mAb was located at 218 aa to 227 aa in the P27 protein in our study. It is worth noting that the 181-227 aa region appears to encompass all of the identified epitopes, suggesting its potential as a prominent B-cell epitope domain. The majority of the domain was exposed on the surface and located within three α-helices (Figure 6C). In addition, the biological information analysis revealed that the epitope was situated on the outer surface of P27, exhibiting a significant antigenic index and strong hydrophilicity. Meanwhile, this region suggested a high likelihood of epitope formation and could more easily be chimeric with antibodies due to its flexibility and ease of twisting and folding. Furthermore, the epitope ^218^IIKYVLDRQK^227^ was highly conserved across all ALV subgroups. These findings enhance our comprehension of the biological attributes associated with the antigen epitope recognized by 1F8 mAb.

Furthermore, the mAb 1F8 was used to develop a sandwich ELISA for detecting ALV antigens. The sandwich ELISA could only react with ALV-A/B/J/K subgroup strains and had high sensitivity, with a LOD of 1.56 ng/mL P27. Compared to a commercial ELISA kit, the sandwich ELISA demonstrated much higher sensitivity in detecting ALV antigens in plasma, semen and cloacal swabs. However, this method did not exhibit improved sensitivity when detecting egg white samples. This might be attributed to the limited exposure of the epitopes in egg white due to its poor fluidity. These results suggest that the application of the mAb-based sandwich ELISA technique may effectively facilitate clinical diagnostics of ALV. Additionally, epitopes play crucial roles in vaccine design and differential diagnoses. Based on the identified epitope of gp85, several epitope-vaccines and peptide-ELISAs have been developed. However, the P27 antibody is ineffective in neutralizing ALV infection. The epitope ^218^IIKYVLDRQK^227^ may not be suitable for epitope vaccine development. Therefore, a peptide-ELISA can be developed to determine ALV antibodies in the future.

In conclusion, a new mAb targeting ALV P27 was successfully prepared and used to develop a more sensitive sandwich ELISA for ALV detection compared to a commercial ELISA kit. In addition, a new linear B-cell epitope, ^218^IIKYVLDRQK^227^, located at the C-terminus of the P27 protein was identified and had a complete homology among all the ALV subgroups strains. These results may be useful for the detection of ALV and carry significant implications for the investigation of ALV protein structure and function.

## Figures and Tables

**Table 1:** Primers used in this study.

Primers	Sequence (5′ to 3′)
P27-F	CAGCAAATGGGTCGCGGATCCCCTGTAGTGATTAAGACAGAGGGACC
P27-R	TGCGGCCGCAAGCTTGTCGACGGCCGCGGCTATGCCTTG
P27-1-F	TTCCAGGGGCCCCTGGGATCCCCTGTAGTGATTAAGACAGAGGGACC
P27-1-R	GATGCGGCCGCTCGAGTCGACATCAGCTAAACCCTTTAAGCGATC
P27-2-F	TTCCAGGGGCCCCTGGGATCCACTAATTTGGATCGCTTAAAGGGT
P27-2-R	GATGCGGCCGCTCGAGTCGACTGGTCCCTGCGTAATGTCCG
P27-3-F	TTCCAGGGGCCCCTGGGATCCGGTCCATGGGCGGACATT
P27-3-R	GATGCGGCCGCTCGAGTCGACGGCCGCGGCTATGCCTTG
P27-3-1-F	TTCCAGGGGCCCCTGGGATCCGGTCCATGGGCGGACATT
P27-3-1-R	GATGCGGCCGCTCGAGTCGACTCGCGCGGAAGGCGGGAG
P27-3-2-F	TTCCAGGGGCCCCTGGGATCCGTTGAGGGGTCAGATCTCCCG
P27-3-2-R	GATGCGGCCGCTCGAGTCGACGGAGGGTGCTGCCCGTAT
P27-3-3-F	TTCCAGGGGCCCCTGGGATCCATACGGGCAGCACCCTCC
P27-3-3-R	GATGCGGCCGCTCGAGTCGACGGCCGCGGCTATGCCTTG
P27-3-3-1-F	TTCCAGGGGCCCCTGGGATCCATACGGGCAGCACCCTCC
P27-3-3-1-R	GATGCGGCCGCTCGAGTCGACCTTCTGCCTGTCTAGCACATATTTG
P27-3-3-2-F	TTCCAGGGGCCCCTGGGATCCATAATCAAATATGTGCTAGACAGGCAG
P27-3-3-2-R	GATGCGGCCGCTCGAGTCGACGGCCGCGGCTATGCCTTG

**Table 2:** Positive samples examined using ELISA and the commercial ELISA kit.

Samples ^a^	Limits of Detection ^b^	
	Sandwich ELISA	IDEXX ELISA
P27 protein	64×	16×
Albumen	32×	32×
Plasma	16×	8×
Semen	64×	32×
Cloacal swab	256×	64×

^a^ Positive samples of ALV detected via PCR. ^b^ Dilution corresponding to the limit of detection.

**Table 3:** Clinical samples examined using ELISA and the commercial ELISA kit.

Sandwich ELISA	IDEXX ELISA					
	Albumen			Cloacal Swabs		
ALV	Positive	Negative	Total	Positive	Negative	Total
Positive	156	3	159	25	14	39
Negative	5	296	301	3	142	145
Total	161	299	460	28	156	184

## Data Availability

All the data are presented in this study.
